# On the neuronal circuitry mediating l-DOPA-induced dyskinesia

**DOI:** 10.1007/s00702-018-1886-0

**Published:** 2018-04-27

**Authors:** M. Angela Cenci, Henrik Jörntell, Per Petersson

**Affiliations:** 1 Basal Ganglia Pathophysiology Unit, Department Experimental Medical Science, Lund University, Lund, Sweden; 2 Neural Basis of Sensorimotor Control, Department Experimental Medical Science, Lund University, Lund, Sweden; 3 The Group for Integrative Neurophysiology and Neurotechnology, Neuronano Research Centre, Department Experimental Medical Science, Lund University, Lund, Sweden; 4 Department of Integrative Medical Biology, Umeå University, Umeå, Sweden

**Keywords:** Movement disorders, Dopamine replacement therapy, Motor systems, Limbic systems, Sensorimotor pathways

## Abstract

With the advent of rodent models of l-DOPA-induced dyskinesia (LID), a growing literature has linked molecular changes in the striatum to the development and expression of abnormal involuntary movements. Changes in information processing at the striatal level are assumed to impact on the activity of downstream basal ganglia nuclei, which in turn influence brain-wide networks, but very little is actually known about systems-level mechanisms of dyskinesia. As an aid to approach this topic, we here review the anatomical and physiological organisation of cortico-basal ganglia-thalamocortical circuits, and the changes affecting these circuits in animal models of parkinsonism and LID. We then review recent findings indicating that an abnormal cerebellar compensation plays a causal role in LID, and that structures outside of the classical motor circuits are implicated too. In summarizing the available data, we also propose hypotheses and identify important knowledge gaps worthy of further investigation. In addition to informing novel therapeutic approaches, the study of LID can provide new clues about the interplay between different brain circuits in the control of movement.

## Introduction

The term l-DOPA-induced dyskinesia applies to abnormal involuntary movements (AIMs) that develop in people with Parkinson’s disease (PD) as a complication of dopamine (DA) replacement therapy. There is vast consensus that such movements depend on the combined effect of DA denervation and standard l-DOPA therapy, ensuing fluctuating levels of DA in the brain and non-physiological stimulation of DA receptors (Carta and Bezard 2011; Cenci 2014). In face of this relatively simple explanation, LID is a complex disorder with variable clinical presentations. Different forms of LID have been described based on the temporal pattern of the involuntary movements relative to medication intake, that is, peak-of-dose dyskinesia, diphasic dyskinesia and off-period dystonia (Fabbrini et al. 2007). Moreover, considerable variability exists on a phenomenological level. In many cases, the involuntary movements are rapid, apparently flowing across body regions in a fast random sequence (chorea). In other cases, the predominant clinical manifestation consists in slow twisting movements and abnormal postures (dystonia). Additional manifestations include abrupt jerky motions and motor stereotypies (Luquin et al. 1992). The pathophysiology underlying these different types of involuntary movements is currently unknown. Here we mainly focus on the most frequent clinical presentation, peak-of-dose dyskinesia. This pattern is readily reproduced in severely DA-denervated animals upon treatment with l-DOPA, usually inducing both hyperkinetic and dystonic components (Cenci and Crossman 2018). In animal models of LID, the expression of involuntary movements is strongly associated with peaks of DA release in the striatum shortly after the administration of l-DOPA (reviewed in Cenci 2014). This is reminiscent of the findings obtained in dyskinetic PD patients using a non-invasive imaging technique to estimate striatal DA release (reviewed in Cenci 2014). Studies in animal models of peak-dose LID have spearheaded much of the recent scientific developments on the topic, as reviewed in this article.

## The striatum

The striatum is the brain structure richest in DA and DA receptors, and disorders of DA transmission have quite a profound impact on information processing in this region (Gerfen and Surmeier 2011; Zhai et al. 2017). On a functional level, the striatum can be viewed as a hub integrating information about internal states and external stimuli to achieve a dynamic and plastic control of motor behaviour. Containing virtually all neurotransmitters so far described in the brain, the striatum is well-suited for these integrative and modulatory functions. The striatum also plays a key role in reinforcement-based learning, the reinforcer being encoded by a phasic burst of firing in DA neurons located in the ventral midbrain (Redgrave et al. 2008), more specifically, in the substantia nigra or ventral tegmental area (VTA) depending on the striatal region considered.

The striatum is the main input structure of the basal ganglia, receiving abundant glutamatergic inputs from the entire cerebral cortex and from the centromedian (CM) and parafascicular (Pf) thalamic nuclei. The response to these excitatory inputs is critically regulated by DA, which modulates both ion conductances and intracellular signaling responses associated with glutamate receptor activation (Gerfen and Surmeier 2011; Nicola et al. 2000; Surmeier et al. 2014). Dopaminergic projections from the ventral midbrain form a dense lattice of axon terminals throughout the striatum, such that all cellular structures within this region are closer than 1 micrometer to a dopaminergic synapse (Moss and Bolam 2008).

**Fig. 1:**
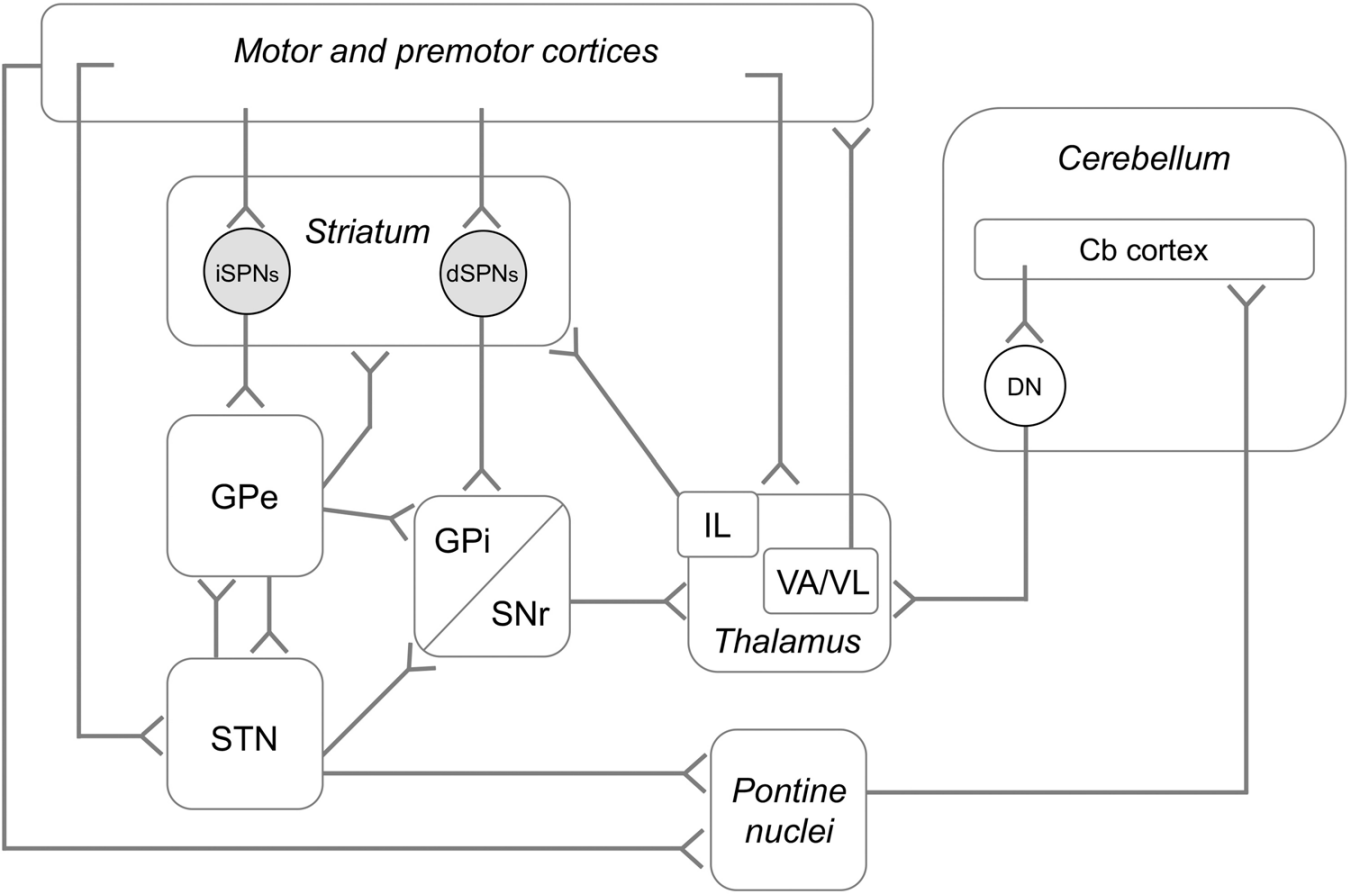
Schematic diagram of cortico-basal ganglia thalamocortical systems and cerebello-thalamocortical systems involved in motor control. The cartoon only illustrates the most important pathways (additional pathways, and axon collaterals of some major ones, are discussed in the main text). *Cb* cerebellar, *DN* deep cerebellar nuclei, *IL* intralaminar thalamic nuclei

The principal type of striatal neurons are the spiny projection neurons (SPNs), often also referred to as medium-sized spiny neurons (reviewed in Gerfen and Surmeier 2011). Two main categories of SPNs convey distinct information to the basal ganglia output nuclei (that is, the substantia nigra pars reticulata, SNr, and the globus pallidus pars interna, GPi) (Fig. 1). “Direct pathway” neurons (dSPNs) project monosynaptically to GPi/SNr, whereas neurons of the “indirect pathway” (iSPNs) influence GPi/SNr via the globus pallidus pars externa (GPe) and the subthalamic nucleus (STN). At the end of the eighties, Albin, Young and Penney proposed the influential hypothesis that the two striatofugal pathways act as a push–pull system to release or inhibit cortically-initiated movements, the two actions being subserved by direct and indirect pathway neurons, respectively (Albin et al. 1989). This hypothesis marked the start of a new era in basal ganglia research because it was seminal to a vast literature searching for distinct roles of striatal output pathways in the modulation of physiological or pathological behaviours. Since dSPNs and iSPNs express distinct complements of membrane receptors and signaling molecules, dissecting the relative contribution of these neurons to parkinsonism and LID appears essential to develop optimal pharmacological therapies (Fieblinger and Cenci 2015).

Striatal neurons forming the direct or indirect pathways are differentially modulated by dopaminergic inputs because they express different types of DA receptors. Thus, dSPNs express G_olf−_ coupled D1 receptors, which both increase the intrinsic excitability of these cells and promote long-term potentiation (LTP) of their glutamatergic synapses. By contrast, iSPNs express the Gi-coupled D2 receptor, which decreases intrinsic excitability and promotes long-term depression (LTD) of glutamatergic synapses (Nicola et al. 2000; Zhai et al. 2017). The striatum also contains an extensive network of GABAergic interneurons and cholinergic interneurons, which play key modulatory roles in all aspects of striatal physiology and behaviour (Tanimura et al. 2017; Tepper et al. 2010). The possible role of striatal interneurons in the pathophysiology of LID is reviewed in other sections of this special issue (Perez et al. 2018).

According to the seminal description of the dual pathway model (Albin et al. 1989), parkinsonism and dyskinesia depend on opposite imbalances in the activity of iSPNs over dSPNs. Thus, iSPN hyperactivity would lead to hypokinesia whereas hypoactivity of the same neurons would lead to dyskinesia via disinhibition or inhibition, respectively, of the basal ganglia output nuclei. Recently, the basic assumptions of Albin and colleagues have been tested experimentally in the mouse using cell type-specific optogenetic or chemogenetic stimulation methods. Overall, these studies have verified the notion that hyperactivity of iSPNs suppresses movement, whereas a large activation of dSPNs can elicit movement (Kravitz et al. 2010) and also lead to dyskinesia (Perez et al. 2017; Hernandez et al. 2017; Alcacer et al. 2017). Using different types of designer receptor exclusively activated by designer drugs (DREADD), Alcacer and colleagues have shown that the mode of dSPN activation is critical to the emergence of abnormal involuntary movements. Thus, dSPN activation via a Gq-mediated signaling pathways improved hypokinetic features in the absence of either axial or limb dyskinesias, whereas Gs-mediated dSPN activation caused dyskinetic movements qualitatively similar to those induced by l-DOPA, even though less severe (Alcacer et al. 2017). On a molecular level, Gs-mediated dSPN activation resulted in a strong stimulation of both extracellular signal-regulated kinases (ERK1/2) and cAMP-dependent signaling, associated with a marked enhancement of action-potential generation in response to depolarizing currents (Alcacer et al. 2017). These and other data (Bateup et al. 2010; Fasano et al. 2010; Heiman et al. 2014; Suarez et al. 2018, 2016) indicate that changes in dSPN activity driven by concomitant strong activation of ERK1/2 and cAMP/PKA signaling are key to the emergence of involuntary movements. Further implicating the critical role of direct pathway overactivity in LID, a recent study has reported that a stable group of dSPNs in the dorsolateral striatum are very active during the expression of dyskinesia, and that optogenetically inhibiting these neurons significantly reduces the severity of LID (Girasole et al. 2018). Notwithstanding the pivotal role of the direct pathway, LID involves pronounced synaptic remodelling of iSPNs (Fieblinger et al. 2014; Suarez et al. 2014, 2018, 2016) and it is improved by chemogenetic stimulation of these neurons (Alcacer et al. 2017). The antidyskinetic effect of iSPN stimulation may depend either on the modulation of pallido-subthalamic activity or on a collateral GABAergic inhibition exerted by iSPNs onto dSPNs at the striatal level. Such collateral inhibition has been proven to underlie the locomotor stimulant effect of cocaine, and has been shown to depend on a stimulation of D2 receptors on the iSPNs (Dobbs et al. 2016).

Considering all the available data, it seems plausible that l-DOPA triggers the expression of abnormal involuntary movements via a D1 receptor-dependent overactivation of pools of dSPNs accompanied by a D2 receptor-mediated inhibition of iSPNs. It also seems plausible that the relative importance of one or the other component differs between subtypes of LID, and this interesting hypothesis clearly deserves further investigation.

## Corticostriatal synaptic plasticity

An important goal in LID research is to understand how the combined effects of DA denervation and l-DOPA treatment lead to altered information processing in the corticostriatal pathway. Almost two decades ago, the hypothesis was put forward that LID represents a pathological form of motor learning sustained by altered corticostriatal synaptic plasticity (Calabresi et al. 2000; Calon et al. 2000). The first direct demonstration of altered corticostriatal synaptic plasticity in LID was provided by Picconi and collaborators using the rat as a model (Picconi et al. 2003). This seminal study reported an association between LID and an inability to reverse long-term potentiation (LTP) of corticostriatal synapses. By applying inhibitors of specific receptors and signaling pathways, the study could link the loss of depotentiation to an excessive activation of D1 receptor-mediated signaling, leading to sustained inhibition of intracellular phosphatases (Picconi et al. 2003). The advent of transgenic mice where the dSPN/iSPN phenotype is reported by fluorescent proteins has opened new possibilities to identify cell type-specific synaptic abnormalities. Exploiting this possibility, Thiele and colleagues applied 6-OHDA lesions and l-DOPA treatment to BAC transgenic mice expressing eGFP in either iSPNs or dSPN. At approximately one month after the lesion, the authors prepared brain slices from these animals and stimulated the corticostriatal pathway using spike-timing dependent plasticity protocols (Thiele et al. 2014). The results of this study show that untreated parkinsonian mice lose the bidirectionality of synaptic plasticity in both SPN types, exhibiting only LTP in iSPNs and long-term depression (LTD) in dSPNs. A therapeutic dose of l-DOPA restored bidirectional plasticity in both SPN subtypes to levels comparable to naïve animals. However, when l-DOPA produced involuntary movements, bidirectional synaptic plasticity was lost again, now with a pattern opposite to that seen in the untreated parkinsonian state. Thus, in the dyskinetic condition, iSPNs only exhibited LTD, whereas dSPNs only showed LTP (Thiele et al. 2014). The authors concluded that a switch from bidirectional to unidirectional plasticity in SPNs underpins both parkinsonism and dyskinesia. By showing that corticostriatal synaptic transmission is enhanced in iSPNs under conditions of hypokinesia and in dSPNs in a hyperkinetic state (LID), these results corroborate the dual-pathway model of basal ganglia pathophysiology proposed by Albin and colleagues (Albin et al. 1989). Albeit interesting, these data should be interpreted with some caution because changes in striatal glutamatergic transmission following DA denervation are time-dependent, and may also depend on the animal model used. Thus, studies in chronically denervated rat have shown that severe DA depletion abolishes both LTP and LTD in all SPNs, although plasticity can be restored by non-dyskinesiogenic regimens of DA substitution, whether provided by cell transplants (Rylander et al. 2013) or l-DOPA administration (Picconi et al. 2003).

## Cortical dynamics

What is currently known about LID-associated cortical dynamics is derived more from human imaging studies than from animal experiments. This situation offers excellent possibilities for back-translating scientific hypotheses from the authentic human disease to experimental models where relevant mechanisms can be dissected at the cellular and molecular level. The first study linking human LID to cortical hyperactivity was based on an analysis of regional cerebral blood flow (rCBF) with single photon emission tomography (SPECT) (Rascol et al. 1998). This study reported that hyperkinetic movements were associated with a significant overactivation of both supplementary motor area and primary motor cortices, attributing this phenomenon to an altered input from basal ganglia-thalamocortical networks (Rascol et al. 1998). The use of rCBF as a surrogate marker of neuronal activity in PD is complicated by the fact that l-DOPA has direct hemodynamic effects in different brain regions, including the motor cortex (Bimpisidis et al. 2017; Jourdain et al. 2016; Ohlin et al. 2012). Nevertheless, the notion that motor cortical areas are hyperactive or dysregulated in LID has now been substantiated by several independent studies based on magnetic resonance imaging (MRI) methodologies (Cerasa et al. 2015; Herz et al. 2015, 2016; Rajan et al. 2017). What remains to be established is the origin of motor cortical hyperactivity “on” l-DOPA in dyskinetic PD patients. Different studies have emphasised different mechanisms, such as, an abnormal functional connectivity between putamen and frontocortical areas (Herz et al. 2015), a dysfunctional coupling between motor areas and inhibitory cortico-cortical networks (Cerasa et al. 2015), altered cerebello-thalamic inputs (reviewed below), or a direct effect of l-DOPA on cortical neurons depleted of their endogenous dopaminergic input (Jourdain et al. 2016). Although several mechanistic questions remain open, changes in functional connectivity and movement-related activity in motor cortical networks can be considered as important components of human LID.

A specific “electrophysiological trait” of LID has been recently revealed by local field potential (LFP) recordings in the motor cortex, detecting pronounced oscillatory activities in large populations of neurons. In a seminal study published in 2012, Halje and colleagues found that high-frequency narrow-band oscillations at ~ 80 Hz in the motor cortex are strongly associated with dyskinetic manifestations in the 6-OHDA rat model of PD/LID (Halje et al. 2012). This strong association was further corroborated by the finding that local pharmacological blockade of D1 receptors in the motor cortex acutely attenuated both the narrow-band 80 Hz oscillations and the abnormal involuntary movements (Halje et al. 2012). By now, the association between high-frequency LFP oscillations and LID has been verified in several independent studies. For example, Judie Walters’ lab has found that the power of cortical 80 Hz oscillations gradually increases as dyskinetic behaviors become more severe during a course of l-DOPA treatment (Dupre et al. 2016). The link between narrowband gamma and LID is currently being explored at several levels (Delaville et al. 2015; Belic et al. 2016; Dupre et al. 2016, 2015; Tamte et al. 2016). Because cortical activity is under strong control of thalamic input, it is possible that an aberrant thalamic input could be driving fast cortical oscillations in LID (Dupre et al. 2015). Altered thalamic input that could push cortical networks into narrowband gamma oscillations would not necessarily consist of an identical oscillatory activity. Preliminary experimental findings, however, suggest that some diffusely projecting thalamic nuclei (which are known to affect cortical states) (Jones 2009) do in fact display coherent oscillations with cortex during LID, and that a local pharmacological suppression of such thalamic activity will eliminate high-frequency oscillations also in the motor cortex (although this suppression was not sufficient to alleviate LID) (Dupre et al. 2015).

Another possibility is that narrowband gamma oscillations are intrinsically generated in the cortical network, from which they spread to several sub-cortical structures. Within the cortical network, intrinsically generated synchronized activity could result from interactions between populations of excitatory principle cells and/or inhibitory interneurons. Depending on the type of oscillation, these cellular sub-groups may be involved to a varying degree. Biologically plausible computer models that incorporate data from *in vitro* intracellular recordings have significantly improved our understanding of how interactions between different cell types contribute to cortical network oscillations (Wang 2010). These studies indicate that an interaction between excitatory pyramidal cells and inhibitory interneurons, or mutual inhibition between inhibitory interneurons, are mechanisms that can support network oscillations, at least in the low gamma range (i.e., ~ 40 Hz) (Jefferys et al. 1996; Whittington et al. 1995). On the other hand, oscillations at very high frequencies (≥ 100 Hz) have been proposed to depend on electrical coupling between fast-spiking interneurons through gap junctions (Galarreta and Hestrin 1999; Gibson et al. 1999).

Recently, Philip Starr’s group published the first long-term cortical recordings in PD patients using an implantable bidirectional device for deep brain stimulation (DBS) and electrocorticography (ECoG) (Swann et al. 2016). This study reports the interesting observation that human LID is accompanied by narrowband high-frequency oscillations in the motor cortex almost identical to those previously described in the rat model. Albeit not as pronounced as in cortex, high-frequency oscillations also occurred in the subthalamic nucleus (STN) (Swann et al. 2016). Although there are previous reports of high-frequency oscillations in the STN during LID (Alonso-Frech et al. 2006; Fogelson et al. 2005), the findings by Swann and colleagues currently represent the strongest evidence for an association between narrowband gamma network oscillations and the expression of abnormal involuntary movements in dyskinetic PD patients.

While more studies are needed in order to prove the causal link between cortical high-frequency oscillations and dyskinesia, this phenomenon provides a valuable biomarker of network dysfunctions that are common to dyskinetic PD patients and animal models of LID. An in-depth dissection of the underlying mechanisms is therefore expected to greatly improve our understanding of LID.

## The pallidal–subthalamic network

The STN and the two segments of the globus pallidus (Fig. 1) form a network of interconnected excitatory and inhibitory neurons within the basal ganglia. Each node of this recurrent network has a distinctive set of afferent and efferent connections. The globus pallidus internal segment (GPi) is the primary target of dSPN projections, and a major component in the output layer of the basal ganglia. As such, the GPi sends inhibitory projections to the motor thalamus and to brainstem nuclei involved in motor control (Fig. 1). The globus pallidus external segment (GPe) receives its two major inputs from STN and striatal iSPNs (Kita and Jaeger 2016). Interesting, however, all dSPNs projecting to the GPi appear to send axon collaterals to the GPe (Wu et al. 2000). These bridging collaterals undergo pronounced plastic changes upon chronic manipulations of D2 receptor activity (Cazorla et al. 2015). Moreover, a significant fraction of subthalamic-pallidal afferents is thought to be shared between internal and external pallidal segments (Koshimizu et al. 2013). All these recent findings have highlighted that the anatomical and functional interactions between GPi and GPe are much more extensive than anticipated by traditional basal ganglia models (Albin et al. 1989; DeLong 1990).

The majority of GPe neurons are GABAergic projection neurons whose main targets are other basal ganglia nuclei (Kita and Jaeger 2016). The output from GPe involves distinct cell groups, that is, prototypical neurons with strong projections to the STN, and the so-called arkypallidal neurons projecting to both SPNs and striatal fast-spiking interneurons (Gittis et al. 2014). Some cells also innervate nuclei of the thalamus, in particular, the parafascicular nucleus (Mastro et al. 2014) and the reticular nucleus (Hazrati and Parent 1991). The latter in turn projects to most of the other thalamic nuclei, being in a position to broadly modulate thalamo-cortical information flow (Nagaeva and Akhmadeev 2006).

Of the above GPe efferents, the most studied one is the robust GPe-STN projection, which has been highlighted as an essential effector of the indirect pathway (Albin et al. 1989; DeLong 1990). In the classical models of basal ganglia organisation, any relative hypo- or hyperactivity of iSPNs is proposed to affect the basal ganglia output nuclei via this GPe-STN pathway (Albin et al. 1989; DeLong 1990). The physiological role of arkypallidal projections has just started to be uncovered (Mallet et al. 2016), and their pathophysiological significance in PD is now being investigated by several laboratories (Gittis et al. 2014).

Being a critical node in the indirect pathway (Albin et al. 1989; DeLong 1990), the STN provides widespread glutamatergic innervation to the basal ganglia output nuclei (the GPi and substantia nigra reticulata, SNr), thus enhancing their inhibitory drive onto motor thalamo-cortical pathways (Fig. 1). The STN is also a critical node in a pathway from motor cortical areas to basal ganglia output nuclei that bypasses the striatum, termed the “hyperdirect pathway” (Nambu et al. 2000). The latter is attributed an important role in response inhibition (Alegre et al. 2013).

The pallidal–subthalamic network is affected by dysfunctions in DA transmission both directly and indirectly. Although cells in the striatum are particularly sensitive to the loss of DA input, practically all parts of the cortico-basal ganglia-thalamocortical network receive some dopaminergic afferents from the midbrain and can potentially sense the fading of DA levels in PD (Rommelfanger and Wichmann 2010). In animal models of PD, the firing of GPe neurons has been reported to be somewhat reduced relative to control conditions (Chan et al. 2011; Filion and Tremblay 1991; Filion et al. 1991), which is in accordance with the classical rate-based models of basal ganglia pathophysiology (Albin et al. 1989; DeLong 1990). In keeping with the rate model are also the findings of increased firing rates in the STN in untreated parkinsonism (Bergman et al. 1990; Remple et al. 2011) and dramatically reduced firing rates in the GPi in LID (Papa et al. 1999). The antidyskinetic effect of GPi lesions (reviewed in Sgambato-Faure and Cenci 2012) is, however, at odds with the rate model. This effect has been taken to indicate that the electrophysiological signature of LID is not so much an overall reduced firing rate but rather an aberrant output signal from the GPi, which would, in turn, disrupt normal patterns of activity in thalamocortical circuits. According to this interpretation, removing the pathological basal ganglia feedback to cortical motor systems would be sufficient to improve both parkinsonism and LID (Brown 2007; Obeso et al. 2009).

## Oscillations in the pallidal–subthalamic network in PD and LID

The advent of DBS as a treatment modality for PD and LID made it possible to carry out LFP recordings from the STN and GPi in human patients, revealing a pronounced association between parkinsonian or dyskinetic motor features and oscillatory activities at specific frequencies (Alonso-Frech et al. 2006; Brown 2007). Oscillations similar to those found in patients were then recorded from both the STN and the basal ganglia output nuclei in both rodent and primate models of PD (reviewed in Bastide et al. 2015; Cenci 2007). These animal models can, therefore be utilised to unveil the neural basis of abnormal oscillatory activities associated with particular motor states. We have already discussed the high-frequency gamma oscillations associated with LID. Below, we will briefly review potential mechanisms of beta-band oscillatory activities in the pallidal–subthalamic network, which are associated with hypokinetic motor states and are reduced by dopaminomimetic treatments (Brown 2007).

In PD, GPe neurons tend to become more synchronized, which could facilitate the generation of pathological oscillations that contribute to motor impairment (Bevan et al. 2002; Brown 2007; Mallet et al. 2008). While beta-oscillations have attracted a lot of interest as a potential mechanism underlying parkinsonism, they differ from LID-linked narrowband gamma oscillations in that beta oscillations are state-dependent and are generally suppressed when the subject is active, whereas narrowband gamma is unaffected by the actuation of voluntary movements (Swann et al. 2016). According to one view, aberrant beta oscillations originate intrinsically in the striatum. Evidence supporting this notion comes particularly from mice in which chronic DA depletion enhances the functional connectivity between striatal fast-spiking interneurons and iSPNs, increasing their synchrony (Gittis et al. 2011). Moreover, acute pharmacological manipulations of the cholinergic striatal interneuronal network has been found to induce striatal beta oscillations (McCarthy et al. 2011). In this context it may be of particular relevance that cells in the indirect pathway of the basal ganglia are easily entrained to oscillatory activity (Sharott et al. 2017), since the reciprocal connections between GPe and STN could maintain and even amplify such network oscillations (Bevan et al. 2002; Plenz and Kital 1999). Other hypotheses suggest that oscillations primarily originate from cortical patterning of striatal and subthalamic activity (Bevan et al. 2006). Both STN and GPe have been shown to fire action potentials coherently with cortical slow oscillations (Magill et al. 2000). In this network, the GPe could enhance STN oscillatory activity by either increasing the capability of the rhythmic excitation from the cortex to drive coherent rhythmic activity in the STN (Baufreton et al. 2005) or through increased transmission of cortical activity to the STN via the striatum (Tseng et al. 2001). It is not known whether this type of network mechanism could also promote much faster gamma oscillations, such as those recorded in the STN in l-DOPA-treated patients (Brown et al. 2001; Swann et al. 2016). But because frontal motor cortices project to both striatum and STN, cortical synchronization can probably entrain at least these two nuclei (which together form the input layer to the basal ganglia, Fig. 1), and animal experiments suggest that such activity can propagate at least to the GPe (Tamte et al. 2016).

## Role of the cerebellum

Accumulating evidence indicates that the basal ganglia and the cerebellum are functionally and anatomically interconnected. The interaction between these brain regions currently represents a hot topic of investigation, not least in the field of movement disorders. In addition, the cerebellum is tightly linked with spinal cord function (Spanne and Jorntell 2013) and the control of brainstem motor nuclei (reticulospinal, rubrospinal, vestibulospinal and tectospinal systems) (Jorntell 2017). These brainstem nuclei have powerful access to the final motor efferents via the spinal cord, and are engaged in all types of movements. Under LID, the cerebellum likely attempts to compensate for a suboptimal movement performance (further discussed below), probably involving the above-mentioned brainstem motor systems in the generation of pathological muscle activation patterns.

It has long been known that the primary motor cortex (M1) receives information from both the basal ganglia and the cerebellum via different parts of the thalamus. In M1, the cerebellar output exerts a physiologically powerful effect (Jorntell and Ekerot 1999). More recently, Peter Strick’s group has uncovered the existence of a two-way communication between the basal ganglia and the cerebellum at the subcortical level. Using retrograde transneuronal viral tracers in non-human primates, Strick’s studies revealed that the dentate nucleus (the largest output nucleus of the primate cerebellum) sends disynaptic projections to the striatum via the thalamus (Hoshi et al. 2005), while the STN projects disynaptically to the cerebellar cortex via pontine nuclei (Bostan et al. 2010). Supporting the functional importance of these anatomical connections, a recent study of STN-DBS in the rat showed that subthalamic high-frequency stimulation significantly decreased neuronal firing not only in the STN, but also in the pedunculopontine nucleus and in cerebellar Purkinje cells. Moreover, costimulation of the STN and the deep cerebellar nuclei at subtherapeutic regimens improved forelimb akinesia to a similar extent as a fully efficacious course of STN-DBS (Sutton et al. 2015).

Evidence for a cerebellar involvement in LID comes from human transcranial magnetic stimulation (TMS) studies. In a series of experiments, Koch and collaborators applied TMS over the lateral cerebellum in a group of dyskinetic PD patients finding that a single session of cerebellar continuous theta burst stimulation (cTBS) transiently reduced LID (Koch et al. 2009). In another experiment, a 2-week course of bilateral cerebellar cTBS induced a persistent reduction of peak-dose LID for up to 4 weeks after the end of the daily stimulation period (Koch et al. 2009). The authors attributed this antidyskinetic effect to a modulation of cerebellothalamocortical pathways. Indeed, cerebellar cTBS was found to reduce short intracortical inhibition and increase long intracortical inhibition, suggesting that the improvement of LID was associated with a reorganization of cortical circuits. Supporting and expanding the above findings, Kishore and collaborators found that deficits in sensorimotor M1 plasticity associated with LID could be reinstated even by a single session of inhibitory cerebellar TMS, and that the antidyskinetic effect of repeated cerebellar TMS was associated with a restoration of sensorimotor plasticity in M1 (Kishore et al. 2014). Based on these results, the authors hypothesized that an abnormal output from the basal ganglia may impact on cerebellar sensory processing, and that the resulting cerebellar dysfunction may contribute to LID by favouring maladaptive plastic responses in M1 (Kishore et al. 2014). Other authors have proposed that the cerebellum is itself an important site of maladaptive plasticity in both PD and LID. According to this view, increased cerebellar activity would be a compensatory response to an altered processing of movement-related information in striato-thalamo-cortical circuits, and robust plastic responses in the cerebellum would increase the likelihood to develop LID (Brusa et al. 2012; Koch et al. 2009).

There is circumstantial evidence of increased cerebellar compensation in LID, as suggested by finding of increased metabolic activity in the deep cerebellar nuclei (Brusa et al. 2012) and in the red nucleus (Lewis et al. 2013), which is an important projection target of the deep cerebellar nuclei.

Based on these and other recent studies (Ferrucci et al. 2016), cerebellar inhibition is now considered as a promising antidyskinetic strategy using either TMS or transcranial direct current stimulation (tDCS) as treatment modalities (Cerasa et al. 2017). Further research is needed to clarify whether and how an abnormal cerebellar compensation contributes to dysfunctional signaling and synaptic plasticity in the striatum and deep basal ganglia nuclei in LID.

## The thalamus

In the influential models proposed by Albin, Penney, Young (Albin et al. 1989) and DeLong (DeLong 1990), the thalamus had a key role in the circuitry generating movement disorders. Indeed, hypokinesia and dyskinesia were attributed to opposite changes (reduction or increase, respectively) in the thalamocortical excitatory drive to motor and premotor areas. Nevertheless, these models did not fit well with the clinical experience from stereotaxic thalamotomies in PD, which improved tremor and rigidity without causing any apparent motor dysfunction (reviewed in Marsden and Obeso 1994). The pathophysiological role of the thalamus in PD and LID continues to be ill-defined, but today we are in a much better position to understand the anatomical, synaptic, and functional features of thalamic nuclei that are embedded in the cortico-basal ganglia-thalamocortical network. We will here review the CM and Pf nuclear complex, then motor thalamic nuclei that represent as a major target of basal ganglia output (Fig. 1).

Located among the caudal intralaminar nuclei of the thalamus, the CM/Pf complex provides the main source of thalamic inputs to the striatum. Thalamostriatal axon terminals of CM/Pf origin form predominantly axodendritic asymmetric synapses with both SPNs and several types of interneurons, particularly the cholinergic ones (reviewed in Smith et al. 2014). In addition to cortical and cerebellar inputs, CM/Pf neurons receive afferents from both the GPi/SNr and a multitude of subcortical nuclei, including the superior colliculus (SC), the pedunculopontine tegmental nucleus (PPN), the raphe nucleus, the locus coeruleus, and several components of the brain stem reticular formation (reviewed in Galvan et al. 2016; Smith et al. 2014). The CM/Pf complex has been recently found to exhibit marked neuronal loss both in PD and in parkinsonian animal models based on neurotoxic DA lesions (Smith et al. 2014; Villalba et al. 2014). This neurodegenerative process occurs early and does not appear to correlate with the severity of parkinsonian motor signs, being instead attributed a causal role in cognitive disturbances (Galvan et al. 2016).

In the classical models proposed at the end of the eighties (Albin et al. 1989; DeLong 1990), the CM/PF complex was lumped together with ventral tier nuclei as being the thalamic target of basal ganglia output. Research in the last decade has revealed specific functions of this nuclear complex in sensorimotor integration and attentional processes that are key to striatum-mediated action selection. For example, electrophysiological studies in the monkey have highlighted the essential role of CM/Pf neurons in the process of attentional orienting to external events occurring on the contralateral side of the body (Minamimoto and Kimura 2002). Consistent with their broad pattern of afferent connections, CM/Pf neurons can respond to a variety of sensory and arousing stimuli, and their activation is believed to favour a switching between actions or attentional sets (Galvan et al. 2016; Yamanaka et al. 2017).

The CM/Pf has recently emerged as an interesting DBS target to treat tics in Tourette’s syndrome (Testini et al. 2016). Within the area of PD and LID, stimulating CM/Pf has potential utility for the management of features that are resistant to standard STN-targeted DBS. Both parkinsonian tremor and disabling dyskinesias have been reported to improve upon CM/Pf-DBS (Caparros-Lefebvre et al. 1999; Stefani et al. 2009). Targeting the CM/Pf complex is, however, considered less effective than standard DBS in relieving hypokinesia and bradykinesia.

The functional impact of CM/Pf high-frequency stimulation in parkinsonian and dyskinetic states has been investigated by Lydia Kerkerian-Le Goff’s group using 6-OHDA hemilesioned rats as a model (Jouve et al. 2010; Kerkerian-Le Goff et al. 2009). Pf-HFS was found to partially ameliorate limb use asymmetry in a test of forelimb hypokinesia while totally reversing lateralized neglect in the so-called corridor test. At the cellular level, Pf-HFS partially reversed the DA denervation-induced increase in striatal preproenkephalin-A mRNA levels (a marker of iSPN dysfunction) without affecting changes in preprotachykinin and preprodynorphin levels (dSPN markers). Pf-HFS reversed the lesion-induced metabolic changes in the STN, GPe, SNr, and partially in the entopeduncular nucleus (i.e., the rodent GPi equivalent). Unlike subthalamic HFS, Pf-HFS did not induce dyskinesias and partially alleviated l-DOPA-induced forelimb dyskinesia (Jouve et al. 2010; Kerkerian-Le Goff et al. 2009). Taken together, these findings reveal that CM/Pf high-frequency stimulation results in a marked amelioration of sensorimotor integration deficits that are associated with dopaminergic lesions. Moreover, CM/Pf stimulation can modulate several basal ganglia nuclei involved in the pathophysiology of LID. On the other hand, CM-selective chemical lesions have been reported not to improve either parkinsonian motor scores or LID in MPTP-lesioned dyskinetic monkeys (Lanciego et al. 2008). Clearly, further investigations are needed to establish the specific contribution of CM/Pf neurons to the pathophysiology of PD and LID.

In all mammalian species, the motor thalamus is consistently located in a ventral region that includes the ventral anterior (VA), ventral lateral (VL) and ventral medial (VM) nuclei. In rats, the anatomical distinction between VA and VL is more difficult to trace, and these nuclei are often considered together as VA/VL (reviewed in Bosch-Bouju et al. 2013). The motor nuclei of the thalamus are a point of convergence between all the motor systems discussed so far, that is, they receive direct input not only from GPi/SNr but also from the cerebral cortex and the deep cerebellar nuclei (Fig. 1). A closer look at the cellular connectivity pattern in VA/VL reveals, however, that the direct convergence between cerebellar and basal ganglia input is rather limited. Indeed, while cortical projections reach all areas of the thalamic motor nuclei, afferents from GPi/SNr reach more anterior regions and remain largely segregated from the cerebellar inputs (which terminates primarily in the posterior part of VL) (Bosch-Bouju et al. 2013). Anatomical species differences exist, and it is worth mentioning that the VM nucleus receives a substantial part of the basal ganglia output in rodents (Bosch-Bouju et al. 2013). Inputs from the cortex (in particular layer V neurons) and deep cerebellar nuclei form large glutamatergic synapses on thalamic neurons, whereas the input from GPi/SNr is exclusively GABAergic.

In the fifties and the sixties, stereotaxic lesions to the motor thalamus were a relatively common intervention for the management of parkinsonism and related conditions. In reviewing the related clinical literature, Marsden and Obeso (Marsden and Obeso 1994) pointed out that thalamic lesions could improve contralateral tremor and rigidity but did not improve nor aggravate hypo/bradykinesia, which was at odds with the prevailing pathophysiological models. More recently, surgical lesions to the motor thalamus have been reported to improve different types of dyskinesia in human patients, including LID (Guridi et al. 2008; Ohye and Shibazaki 2001). These results support the hypothesis that an increased activity of motor thalamic nuclei (secondary to changes in basal ganglia output) plays a causal role in the genesis of hyperkinetic movements (Albin et al. 1989; DeLong 1990). Further investigations are needed to unravel how an altered processing of basal ganglia signals in the motor thalamus contribute to parkinsonian or dyskinetic states.

## The newly discovered role of some unconventional nuclei

In animal models of PD, the development of LID is associated with pronounced transcriptional changes in striatal projection neurons, among which, the upregulation of transcription factor delta-FosB was the first to be discovered (Andersson et al. 1999) and was later detected also in the post-mortem putamen of LID patients (Lindgren et al. 2011). Recently, Bastide and collaborators mapped the expression of deltaFosB and other immediate-early genes (IEGs) across the entire brain in the rat model of LID (Bastide et al. 2014). When comparing dyskinetic to non-dyskinetic animals, these authors found a striking increase in IEG immunoreactivity in several regions not directly involved in motor control, such as, the bed nucleus of the stria terminalis, the dorsal hippocampus, the zona incerta, and the lateral habenula (Bastide et al. 2014). Prompted by these observations, Bastide and colleagues applied a new pharmacogenetic method to selectively inactivate the neurons expressing delta-FosB in the lateral habenula (Bastide et al. 2016) or in the bed nucleus of the stria terminalis (Bastide et al. 2017). In both cases, the local neuronal inactivation yielded a significant antidyskinetic effect. Bastide and colleagues attributed the l-DOPA-induced upregulation of delta-FosB in these two unconventional nuclei rather to a local expression of D1 receptors than to circuit mechanisms. It now remains to be established how a selective inactivation of dopaminoceptive neurons in either of these two nuclei can influence the motor circuits mediating LID. Both the lateral habenula and the bed nucleus of the stria terminalis are part of limbic systems involved in affective and emotional control (Baker et al. 2016; Dumont 2009; Yang et al. 2017). Based on previous metabolic mapping studies in NHP models, Erwan Bezard and collaborators had indeed proposed that LID engages also limbic and associative domains of the basal ganglia, and not simply the motor ones (Guigoni et al. 2005). Although little is known about the role of limbic circuits in LID, emotional states appear to have an impact on the expression and severity of involuntary movements. By observing rodent model of LID, we have noticed that the abnormal involuntary movements induced by l-DOPA are more severe in situations perceived as stressful by the animal (unpublished data from the Cenci lab). Similar observations are often reported by the investigators involved in clinical trials of putative antidyskinetic drugs, where context-dependent variations in LID severity can be a significant confounding factor. These considerations highlight the need for further research to unravel how emotional states encoded in limbic regions influence the operations of motor effector pathways in both healthy and diseased states.

## Concluding remarks

It is both fascinating and medically important to unravel network dysfunctions at the basis of LID. In addition to improving our understanding of the brain motor circuitry, this research will allow for defining precise therapeutic targets for antidyskinetic treatments based on either electrical or pharmacological methods. Moreover, an improved understanding of the interplay between motor, limbic, and cognitive circuits in LID may inspire entirely novel strategies of symptom control, akin to multifunctional training and cueing approaches that are currently being explored for other symptomatic domains in PD (Mak et al. 2017; te Woerd et al. 2015).
